# The Frequency of Gall Stones

**Published:** 1901-12-07

**Authors:** 


### THE FREQUENCY OF GALL STONES.

There are certain conditions which are much more frequently discovered by the pathologist than by the physician, and among such is the existence of gall stones. Many a patient suffers for years from more or less ill-defined ailments which are put down to chronic dyspepsia, constipation, billiousness, or what not, and the real origin of which is never discovered until perhaps the pathologist finds a gall-bladder full of calculi. Even then it may be impossible to speak with certainty as to the relationship between the gall stones and the maladies, but, at least, it may be stated that, in many cases, if it could have been known during life that these stones were present, they would almost certainly have been regarded as the probable explanation of the symptoms exhibited by the patient If we did but recognise the unreliability of the generally accepted symptoms of gall stones and the frequency with which these concretions exist without showing any of the recognised signs of their presence, we should probably be much more ready than we are to think of gall stones in all cases of abdominal trouble associated with painful indigestion. Dr. Fiedler[1] has lately stated that out of 93,000 patients examined, gall stones were noted during life in only 133, *i.e.*, in 0·14 per cent., while at autopsies gall stones are found in fully 10 per cent. of the bodies examined if a careful examination of the biliary system be made. The difference between the frequency of the *ante-mortem* and the *post-mortem* discovery of gall stones is very striking, and shows in how many cases this possible cause of many troubles remains undiscovered and unsuspected. Not that we would suggest that the gall stones are the cause of all the abdominal symptoms which occur in cases where they exist, for, indeed, there is much reason for believing that even before a gall stone forms there has been some preceding catarrh or disorder of the biliary passages. Nor can one say, as some might perhaps suggest, that these figures show in how large a number of cases treatment ought to have been surgical rather than medical, for even after removal of gall stones recurrence takes place in a not inconsiderable proportion of the cases. What these figures really show is that biliary catarrh, even in its slighter forms, is a more serious affair and more common complaint than some have imagined, and that in cases in which there is reason to believe that it may have occurred, treatment should not be confined to the mere relief of the symptoms which have produced distress, but should be so long continued and should take such a direction as to restore the healthy action of the liver and thus prevent the formation of biliary calculi.

[1] Münch. Med. Woch., Oct. 25.

